# Spectral characteristics of light and their association with myopia progression in children: A systematic review protocol

**DOI:** 10.1371/journal.pone.0355383

**Published:** 2026-08-20

**Authors:** Gabriel Kwaku Agbeshie, Isaiah Osei Duah Junior, Albert Kwadjo Amoah Andoh, Josephine Ampong, Nana Akwasi Owusu Mensah, Awurama Yenkua Ampoma-Mensah, Eunice Acheampong Boadu, Debora Baidoo, Kwadwo Owusu Akuffo

**Affiliations:** 1 Department of Optometry and Visual Science, College of Science, Kwame Nkrumah University of Science and Technology, Kumasi, Ghana; 2 Department of Psychology, John R. and Kathy R. Hairston College of Health and Human Sciences, North Carolina Agricultural and Technical State University, Greensboro, North Carolina, United States of America; 3 Galaxy Eye Care Limited, Sekondi-Takoradi, Ghana; Tehran University of Medical Sciences, IRAN, ISLAMIC REPUBLIC OF

## Abstract

The rising prevalence of myopia, particularly among children, has emerged as a major global public health concern. Emerging evidence indicates that the spectral composition of light may play a critical role in regulating ocular growth and influencing the onset and progression of myopia. However, the available evidence is characterized by substantial heterogeneity in study design, exposure assessment, and myopia-related outcome measures, making it difficult to draw consistent conclusions. This systematic review aims to identify and evaluate specific spectral profiles of light associated with measurable reductions in myopia progression. To achieve a comprehensive synthesis, evidence from observational human studies and clinical trials will be systematically reviewed. A systematic literature search will be conducted across major databases including PubMed, Scopus, Web of Science, Embase, Cumulative Index to Nursing and Allied Health Literature (CINAHL), PsycINFO, Cochrane Register of Controlled Trials (CENTRAL), and Google Scholar, without restrictions on language or publication period using defined keywords. Eligible studies will include those examining the relationship between light wavelength, spectral irradiance, temporal dynamics, and myopia progression. Two reviewers will independently perform study selection, data extraction, and quality appraisal using validated tools appropriate to each design, including the Cochrane Risk of Bias 2 (RoB 2), Risk of Bias in Non-Randomized Studies of Interventions (ROBINS-I), Joanna Briggs Institute (JBI) checklists, and the National Heart, Lung, and Blood Institute (NHLBI) quality assessment tools. Data will be synthesized narratively, and a meta-analysis conducted where study comparability permits. Taken together, this systematic review protocol outlines a rigorous and transparent approach to consolidate current evidence on the influence of light spectra on myopia development, identify methodological limitations, and outline research priorities to guide future clinical trials, and public health strategies aimed at mitigating childhood myopia progression globally. This review is registered in PROSPERO [CRD420251171027].

## Background

Myopia, commonly known as nearsightedness, is a refractive error in which distant objects appear blurry [1]. Over the past three decades, the global prevalence of myopia, particularly among children, has increased dramatically [2]. This trend has transformed myopia from a simple refractive inconvenience into a major public-health concern. The growing incidence of high myopia, in particular, poses long-term risks for irreversible, sight-threatening complications such as retinal detachment, myopic maculopathy, and glaucoma [3–5]. Recent global estimates indicate that myopia now affects an unprecedented proportion of children and adolescents, with projections suggesting that nearly half of the world’s population may be myopic by 2050 [3]. These projections highlight the urgent need for evidence-based interventions that can be implemented early in life to slow myopia progression and reduce the risk of developing high myopia and its associated sight-threatening complications.

A consistent and well-replicated finding in epidemiological research is that increased time spent outdoors reduces the risk of myopia onset in children [6–9]. This protective effect has been observed across multiple populations, age groups, and study designs, suggesting a robust environmental influence on ocular development. Traditionally, researchers have attributed this relationship primarily to higher ambient light intensity in outdoor settings compared with indoor environments [7,10–14]. However, sunlight differs from artificial indoor lighting in more than just brightness. It also varies in spectral composition (distribution of light across different wavelengths), temporal dynamics (the pattern and fluctuation of light exposure throughout the day), and spectral irradiance (the power of specific wavelength bands reaching the retina). These distinct optical characteristics imply that the quality of light, rather than its quantity alone, may play a key role in regulating ocular growth and refractive development.

Recent advances in visual neuroscience and experimental ophthalmology have uncovered biologically plausible mechanisms that link the spectral composition of light to ocular growth regulation [15]. Evidence from animal studies indicates that light of different wavelengths can modulate retinal neurochemistry, particularly dopamine release, a critical neuromodulator in eye growth regulation [16–19]. Dopamine acts as a “stop” signal for excessive axial elongation, and its release is sensitive to both light intensity and wavelength. Other studies have demonstrated that chromatic cues derived from the differential focusing of long and short wavelengths on the retina contribute to emmetropization, the eye’s natural process of achieving optical focus during growth [20–25]. Furthermore, variations in spectral exposure may alter retinal contrast sensitivity and downstream signaling pathways that influence scleral remodeling, collectively shaping axial growth patterns.

These mechanistic insights have stimulated an expanding body of human research examining how specific spectral bands of light might influence myopia development or progression. Several clinical and observational studies have investigated interventions or exposures designed to manipulate spectral content. For instance, studies of violet-light exposure [22,26–31], blue-light–enriched environments [31–40], and repeated low-level red-light (LLRL) therapy [41–65] have sought to determine whether targeted wavelength exposure can modulate ocular growth in children. Among these, randomized and prospective trials evaluating repeated low-intensity long-wavelength (red) light therapy have reported promising outcomes, including significant reductions in both axial elongation and refractive progression in school-aged children [64,66–69]. Meanwhile, other investigations have suggested potential protective roles for short-wavelength (blue or violet) light and broad-spectrum sunlight, which may enhance retinal dopamine activity or provide chromatic signals that support emmetropization [20,32].

### Rationale for this review

Despite these promising ascertions, results across studies remain heterogeneous. Some evidence suggests that overall illuminance, that is, the total intensity of light exposure, or temporal exposure patterns (duration, frequency, and timing of light exposure) may be more influential than spectral characteristics alone [70]. Methodological differences in study design, sample characteristics, exposure measurement techniques, and outcome metrics (such as axial length versus refractive error) further complicate the synthesis of results [14]. Consequently, while the potential role of spectral composition in myopia control is increasingly recognized, the field lacks a comprehensive synthesis that systematically evaluates the strength and direction of these associations.

Given the rapid translation of spectral-based findings into clinical and commercial applications, including red-light therapy devices, spectrally tuned indoor lighting systems, and violet-transmitting spectacle lenses, a critical, evidence-based appraisal of this emerging literature is both timely and necessary. A systematic review, with meta-analysis where appropriate, is needed to synthesize the available evidence on the relationship between the spectral properties of light and myopia progression in children and to evaluate whether specific light wavelengths are associated with clinically meaningful reductions in myopia progression. Moreover, such a review will identify key sources of heterogeneity, including differences in study populations, exposure definitions, follow-up durations, and outcome measures. Beyond its academic significance, understanding the influence of light’s spectral composition has direct implications for public eye health, industry, and technology. If particular wavelengths are shown to protect against myopia progression, this knowledge could inform practical, non-pharmacological interventions, such as optimizing classroom lighting, promoting safe outdoor exposure, development of wavelength-specific optical devices, smart lighting systems, and display technologies. These advances could support evidence-based innovations in the lighting and vision care industries while contributing to strategies aimed at slowing the global rise of childhood myopia.

To address this knowledge gap, this systematic review aims to answer the primary question: “What is the relationship between the spectral properties of light and myopia progression in children?” Specifically, the review will address the following questions: 1) Which wavelengths or spectral compositions of light are associated with reduced or increased myopia progression in myopic and premyopic children? 2) What is the magnitude of the effect of different light spectra on clinically relevant myopia outcomes (axial length elongation, spherical equivalent refractive error)? 3) How do variations in study characteristics such as population demographics, exposure definitions, follow-up duration, and outcome measures contribute to heterogeneity in reported associations? 4) What evidence exists to support the translation of spectral light exposure into clinically relevant interventions for myopia control in children? Through systematic synthesis of the available evidence, this review will characterize current knowledge, identify methodological gaps, and provide a foundation for future clinical trials, translational innovations, and preventive eye-care strategies informed by the photobiology of ocular growth.

## Materials and methods

**Fig 1:**
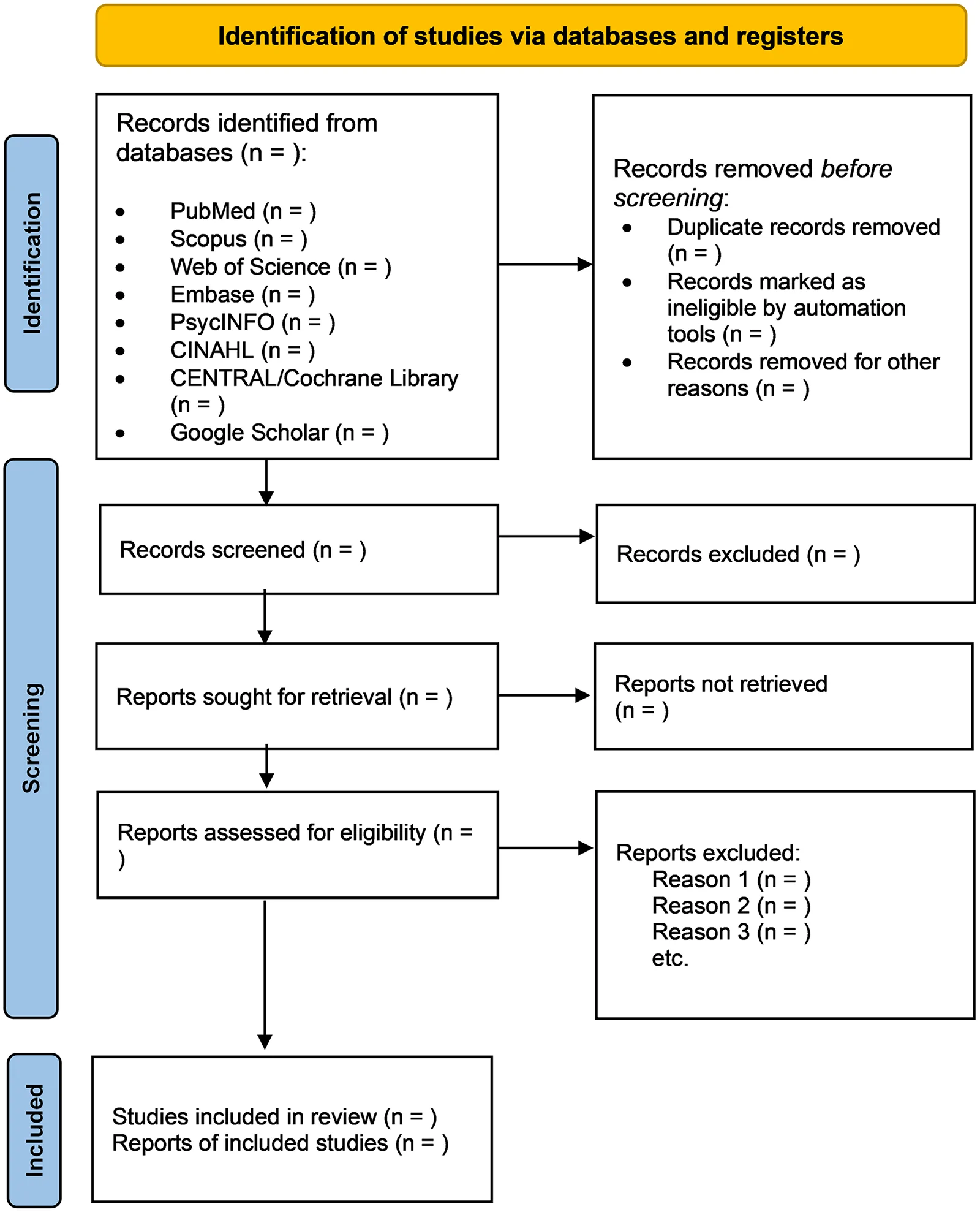
PRISMA flow diagram illustrating the study selection.

This review protocol (PROSPERO ID: CRD420251171027) has been developed in accordance with the Preferred Reporting Items for Systematic Review and Meta-Analysis Protocols (PRISMA-P) guidelines (S1 Table) to ensure methodological rigor and transparency [71,72]. The completed review will adhere to the PRISMA reporting standards [73,74] and follow the recommendations outlined in the Cochrane Handbook to promote robustness and reproducibility [75]. The primary aim of this systematic review is to synthesize current evidence on the relationship between the spectral characteristics of light exposure and myopia progression in children. Specifically, it will assess whether particular wavelengths or spectral profiles, such as short-wavelength (blue-enriched), long-wavelength (red-enriched), or broad-spectrum (sunlight-like) light, are associated with clinically meaningful reductions in myopia progression. In addition, the review will explore methodological sources of heterogeneity, including variations in study populations, exposure metrics, outcome measures, and follow-up durations. Through robust analysis framework, this review aims to identify key knowledge gaps and inform the design of future clinical trials and public eye health recommendations. The protocol details the planned methodology, encompassing the search strategy, eligibility criteria, data extraction procedures, risk of bias assessment, and data synthesis approach. The process of study identification and selection will be systematically documented using a PRISMA flow diagram (see Fig 1). By adhering to this pre-registered and methodologically structured protocol, the review minimizes the potential for reporting bias, enhances transparency, and ensures that all methodological decisions are pre-specified, thereby strengthening the reliability of conclusions regarding the role of spectral light characteristics in childhood myopia progression.

### Eligibility criteria

#### Population (P).

Studies will be eligible for inclusion if they examine children (5–12 years) and adolescents (13–18 years) irrespective of sex, racial background, and ethnic origin or degree of myopic refractive error (i.e. myopia or premyopia of any degree). Myopia will be defined according to the International Myopia Institute (IMI) as a spherical equivalent refraction (SER) of ≤ −0.50 diopters (D) of an eye when ocular accommodation is relaxed whereas premyopia will be defined as a refractive state of an eye of ≤ + 0.75 D and > −0.50 D in children, where a combination of refractive error, age, and other risk factors indicates a sufficient likelihood of developing myopia to justify preventive interventions [76]. Studies that include mixed-age populations will be considered only if data specific to the paediatric subgroup can be extracted separately to allow for valid comparisons and synthesis.

#### Intervention/Exposure (I).

The intervention and/or exposure of interest is light with defined spectral characteristics. Eligible studies may involve both natural and artificial sources such as sunlight, LED, fluorescent, incandescent, and screen-based lighting. To qualify for inclusion, studies must report quantifiable or defined spectral compositions such as blue-enriched, red, or violet light or otherwise to characterize the wavelength distribution of the light exposure. Both controlled experimental interventions and observational exposures assessing the influence of specific light spectral profiles on myopia progression will be included. For quantitative synthesis, studies will be grouped according to comparable spectral exposure categories, intervention characteristics, and outcome measures where sufficient data are available.

#### Comparator (C).

Comparators will include alternative light spectra, conventional indoor/artificial lighting, lower light exposure conditions, no specific spectral light exposure, usual lighting conditions, or sham exposure conditions. Comparator definitions will be considered according to the study design and exposure context to facilitate appropriate evidence synthesis.

#### Outcomes (O).

The primary outcomes of interest are measures of myopia progression, specifically change in spherical equivalent refraction (SER) and/or axial elongation (AXL). Studies that report both outcomes will be prioritized for synthesis. Secondary outcomes may include parameters directly related to mechanisms of myopia control, such as changes in choroidal thickness, provided that these are accompanied by SER or AXL data.

#### Study design (S).

Eligible study designs will include randomized controlled trials (RCTs), and observational studies (i.e. cohort studies, case-control studies, and cross-sectional studies) that provide data on light spectral properties and myopia progression. These designs are expected to provide sufficient methodological rigor and data consistency to address the review objectives.

### Exclusion criteria

Studies will be excluded if they involve adults older than 18 years only or if paediatric data cannot be extracted from mixed-age samples. Studies will be excluded if they focus exclusively on optical or pharmacological myopia-control interventions unrelated to light spectral exposure, including spectacles, contact lenses, orthokeratology, or atropine therapy, without evaluating the spectral characteristics of the light environment or exposure.Similarly, studies will be excluded if they do not report or define the spectral characteristics of light exposure or if they assess only visual acuity, accommodative lag, or choroidal thickness without corresponding measures of refractive error or axial length.. All forms of reviews, editorials, letters, opinions, and case reports will also be excluded. Additionally, preprints and conference abstracts that have not undergone peer review will be excluded.

### Data sources, search terms, and search strategies for identifying studies

Electronic databases to be systematically searched will include PubMed, Embase, Web of Science Core Collection, Scopus, the CINAHL, PsycINFO, CENTRAL. The search will cover all records from database inception to the date of search execution. To further enhance coverage, manual searches will be performed on the first ten pages of Google Scholar, and reference lists of included articles and related systematic reviews will be screened to identify any additional eligible studies.

Given that the selected databases index studies published in multiple languages, no language restrictions will be applied during the search. Where non-English articles are identified, artificial intelligence–assisted translation tools, including large language model (LLM)-based translation approaches where appropriate, will be used to facilitate initial screening and data extraction. Translations and extracted data will be verified by multilingual researchers whenever feasible to ensure accuracy and minimize misinterpretation. The search will not be limited by publication date. The search strategy will be organized according to the PICOS framework – Population, Intervention, Comparison, Outcomes, and Study Design. Population terms will focus on children with myopia. Intervention or exposure terms will relate to spectral light characteristics. Outcomes will include myopia progression, changes in spherical equivalent refraction, axial elongation, and other refractive error changes. Study design terms will capture randomized controlled trials and observational designs.

Boolean operators (AND, OR) and truncation symbols will be used to combine related keywords, and appropriate database-specific indexing terms – such as MeSH in PubMed and Emtree in Embase – will be applied. Search terms would include (“myopia” OR “nearsightedness” OR “short-sightedness”) AND (“light exposure” OR “illumination” OR “wavelength” OR “spectral composition” OR “light spectrum” OR “blue light” OR “violet light” OR “red light” OR “natural light” OR “artificial lighting”) AND (“children” OR “adolescents” OR “school-aged” OR “pediatric”) AND (“myopia progression” OR “axial elongation” OR “AXL” OR “refractive error change” OR “spherical equivalent refraction” OR “SER”). Filters may be applied to identify both clinical and experimental studies examining myopia progression in response to specific light spectra.

All search strategies will be developed iteratively and independently reviewed by two researchers to ensure completeness and precision. The final search strings for each database will be documented in the review report to promote transparency, reproducibility, and adherence to systematic review standards.

### Study selection

All search results will be exported into EndNote for compilation. The references will then be imported into the Covidence systematic review management platform for screening and review. Two independent reviewers (G.K.A. and J.A) will screen titles and abstracts to identify potentially eligible studies based on predefined inclusion and exclusion criteria. Any disagreements during this stage will be resolved through discussion, and a third reviewer (I.O.D.J) will arbitrate unresolved discrepancies. Full-text screening will then be conducted independently by the two reviewers using the eligibility criteria. Reasons for excluding studies at the full-text stage will be carefully recorded, and the overall selection process will be summarized in a PRISMA flow diagram.

### Data extraction

Data from included studies will be extracted using a standardized and piloted data extraction form designed specifically for this review. Extracted variables will include general study information (author, year of publication, country, and study design); participant characteristics (sample size, mean age, sex distribution, ethnicity, and myopia baseline status); and exposure details (light source, including natural broad-spectrum sunlight versus narrow-spectrum indoor artificial lighting (e.g., LED, fluorescent, incandescent); spectral characteristics of light (wavelength or spectral composition (e.g., violet, blue, red, or full-spectrum light), spectral irradiance or spectral power distribution, illuminance intensity (lux), duration of exposure, frequency of exposure, timing of exposure (e.g., daytime versus evening); long-term safety outcomes of spectral exposure (structural and functional vision, patient-reported symptoms); near work activities (working distance, viewing duration, task type, and frequency of breaks); baseline myopia risk factors (parental history of myopia, visual task and environment, age at myopia onset, refractive status). Information regarding whether and how these potential confounding variables were measured and statistically adjusted for in the original studies will also be recorded. Outcome-related data will cover myopia progression measures such as changes in spherical equivalent refraction, axial elongation, and rate of refractive change over time, as well as follow-up duration and analytical approach. Quantitative data, including effect sizes, standard deviations, and confidence intervals, as well as any relevant qualitative findings, will be extracted. Funding sources and potential conflicts of interest will also be noted to assess study bias. Two reviewers will independently perform data extraction (G.K.A and J.A), and discrepancies will be resolved through discussion and consensus by a third reviewer (I.O.D.J).

### Risk of bias

The risk of bias of all included studies will be independently assessed by two reviewers (G.K.A and J.A) using validated tools appropriate for the respective study designs. Randomized controlled trials (RCTs) will be assessed using the Cochrane Risk of Bias 2 (RoB 2) tool [77], while non-randomized or quasi-experimental studies will be evaluated with the ROBINS-I tool [78]. For observational studies, such as cohort or cross-sectional designs, the Joanna Briggs Institute (JBI) critical appraisal checklists [79] will be used. These appraisal tools would be used to assess confounding bias in the selected studies. Particular attention will be given to whether included studies adequately controlled for major environmental and behavioral confounders relevant to myopia progression. Any discrepancies between reviewers will be discussed and resolved through consensus, with arbitration by a third reviewer (I.O.D.J) when necessary.

### Quality of assessment

To ensure a comprehensive evaluation of study quality, the National Heart, Lung, and Blood Institute (NHLBI) Study Quality Assessment Tools [80] will be used across relevant study designs. These tools will help assess the internal validity, methodological soundness, and potential sources of confounding bias in each study. Each included study will be rated as good, fair, or poor quality based on predefined criteria, including clarity of research objectives, adequacy of sample size, appropriateness of spectral exposure characteristics measurement (light wavelength or spectral composition, illuminance intensity, exposure duration, and spectral power distribution), near work activities, accuracy of myopia outcome assessment (e.g., axial length or refractive change), statistical robustness, and adjustment for confounding factors such as age, time spent outdoors, and temporal exposure patterns. The integration of these appraisal tools will ensure that the credibility and reliability of the evidence are rigorously evaluated. Any disagreements in quality ratings will be resolved through discussion or consultation with a third reviewer, and the final quality assessments will be reflected in the narrative synthesis and interpretation of the review findings.

### Data synthesis

For heterogeneity in study designs, population characteristics, exposure definitions, and myopia outcome measures across included studies, a narrative synthesis will primarily be employed to summarize the evidence and, where feasible, through subgroup analyses. The synthesis will be structured around key dimensions such as study design (randomized, observational, or experimental), population characteristics (age, baseline myopia level, geographic region, and ethnicity), spectral exposure characteristics (light wavelength or spectral composition, illuminance intensity, spectral power, duration and timing of light exposure and light source type), and outcome measures (change in spherical equivalent refraction, axial elongation, or refractive error progression rate). Patterns of association will be explored, and methodological factors contributing to heterogeneity, such as measurement techniques, exposure quantification, and follow-up duration, will be highlighted.

Where the data characteristics of retrieved studies are sufficiently comparable (i.e., at least two studies with similar exposure definitions and outcome measures), a meta-analysis will be conducted using a random-effects model to account for between-study variability. Effect sizes (e.g., mean difference or standardized mean difference for continuous outcomes) and their 95% confidence intervals (CIs) will be calculated. Statistical heterogeneity will be quantified using the I² statistic, with values of 25%, 50%, and 75% representing low, moderate, and high heterogeneity, respectively. If adequate data are available, subgroup analyses and contextual interpretation will be performed based on wavelength range (e.g., full-spectrum, narrow-spectrum), light source (natural vs. artificial), geographic region, ethnicity or study design and behavioral and environmental myopia risk factors to assess the consistency and generalizability of the evidence. Interpretation of spectral effects will therefore consider the extent to which confounding variables were measured and adjusted for within individual studies. Sensitivity analyses will be carried out by excluding studies rated as high risk of confounding bias. Contrary, synthesis without meta-analysis (SWiM) will be adopted supposed retrieved data are heterogeneous [81].

### Publication bias

If a meta-analysis includes at least ten studies, publication bias will be assessed visually using funnel plots and statistically using Egger’s regression test or Begg’s rank correlation test to detect potential small-study effects.

### Confidence in cumulative evidence

The overall certainty of evidence across included studies will be assessed using the Grading of Recommendations Assessment, Development, and Evaluation (GRADE) framework. This approach will evaluate the strength and reliability of the evidence for each outcome related to myopia progression (e.g., change in spherical equivalent refraction and axial elongation) in relation to spectral light exposure. The evidence will be rated as high, moderate, low, or very low certainty based on factors such as risk of environmental and behavioral confounding bias, consistency of results across studies, directness of evidence, precision of estimates, and potential publication bias. The GRADE assessment will provide a transparent summary of the confidence in cumulative evidence, thereby guiding the interpretation of the findings and their relevance for clinical and public health recommendations.

### Handling missing data

When studies present incomplete, unclear, or missing data, the review team will attempt to contact corresponding authors to obtain clarification or missing information. If these data cannot be retrieved, the extent and type of missing information will be clearly documented. For quantitative analyses, the potential influence of missing data on effect estimates will be carefully considered. Where possible, sensitivity analyses will be performed to determine how assumptions about missing data affect the overall results. Studies with substantial missing data that may compromise validity will either be rated as lower quality or excluded from quantitative synthesis, while their findings will still contribute to the narrative synthesis. Missing data considerations will also inform the risk of bias assessment using tools such as the RoB 2, ROBINS-I, JBI, SYRCLE and NHLBI quality appraisal tools. This structured approach will help minimize bias and ensure conclusions are based on the most complete and credible evidence available.

### Ethical consideration and dissemination

This study is a systematic review protocol that involves the synthesis of data obtained exclusively from previously published studies and publicly available sources. As no new human participants or animals are directly involved, and no identifiable personal data are collected or analyzed, ethical approval is not required. All data included in the review will be extracted from studies that have received prior ethical clearance as stated by their respective authors. The review will be conducted in accordance with established ethical standards for secondary research and reporting guidelines such as PRISMA. Of note, the results that emanates from this review will be disseminated through peer-reviewed journals, conference presentations, and academic symposia focused on myopia, vision science, and ocular epidemiology. In addition, findings will be shared with researchers, clinicians, and public health stakeholders to inform future experimental studies, clinical guidelines, and environmental recommendations related to light exposure and myopia control in children.

### Timeline and status of review

This study protocol was developed in November 2025 with the optimization of search strategy and its registration in PROSPERO (CRD420251171027). We aim to complete this review in 8 months with literature screening (title/abstract and full-text screening) to take place in August 2026 with respect to feedback from the peer reviewers. Data extraction is expected to be completed by October 2026, with the timeline dependent on the number of included studies. Evidence synthesis, interpretation of findings, and manuscript drafting are expected to be completed by December 2026 (S2 Table).

## Discussion

This proposed systematic review aims to address a critical gap in the current literature by synthesizing evidence on how human studies have advanced understanding of the spectral characteristics of light associated with clinically meaningful reductions in myopia progression. Myopia has become a major global public health challenge, with prevalence rising sharply among children and adolescents [82,83]. Although growing evidence indicates that light exposure plays an important role in ocular growth regulation [15,84,85], the precise wavelength ranges that exert protective effects against myopia remain incompletely characterized. By systematically consolidating evidence fromhuman centered studies among children, this review seeks to identify specific spectral profiles that may impede myopia development and to assess the consistency and strength of the evidence supporting such associations.

A key strength of this review is its comprehensive and structured approach, guided by PRISMA and Cochrane recommendations. The inclusion of both interventional and observational human studies will allow for a broad synthesis of available evidence, capturing real-world exposure contexts and diverse population settings. Furthermore, the review will systematically evaluate methodological factors that may influence interpretation of wavelength-specific effects, including illuminance intensity, exposure duration, outdoor activity, near work, and temporal exposure patterns.

However, some methodological and practical limitations are anticipated. First, the heterogeneity across included studies, particularly in design, population characteristics, and measurement techniques, may pose challenges for meta-analysis. Differences in defining and quantifying light exposure (e.g., wavelength bands, light source types, or exposure duration) and variability in myopia-related outcomes (e.g., axial length versus spherical equivalent refraction) may limit direct comparability. Importantly, a major anticipated challenge will be disentangling the independent effects of spectral composition from other highly interrelated environmental and behavioral factors associated with myopia progression. Variability in how studies measure and adjust for confounding variables such as outdoor exposure, near work activities, baseline myopia risk, and overall light intensity may reduce the ability to draw causal inferences regarding wavelength-specific effects. Consequently, findings will be interpreted cautiously with emphasis placed on methodological rigor, confounder adjustment, and consistency of associations across studies. Second, publication bias, especially the tendency to report significant or positive findings, could distort the overall conclusions. Third, the potential scarcity of high-quality randomized controlled trials focusing on spectral effects may restrict causal inference. In addition, although we will consider evidence beyond English-language publications during screening, translation and interpretation will be constrained by available institutional resources. Further, the evidence base may be disproportionately represented by East Asian populations, which could limit the generalizability of findings to other ethnic and geographic settings. Despite these limitations, this systematic review is expected to yield valuable insights into the role of light spectral characteristics in myopia progression. By identifying consistent patterns across studies and highlighting gaps in current evidence, it will establish a stronger empirical foundation for the design of future controlled trials and inform evidence-based public health strategies. Collectively, the synthesis will contribute to refining myopia prevention frameworks that integrate biological plausibility with practical implementation across diverse environmental and population contexts.

## Supporting information

S1 Table PRISMA-P (Preferred Reporting Items for Systematic review and Meta-Analysis Protocols) 2015 checklist. (PDF)

S2 Table Timeline and status of review. (PDF)

## Abbreviations

CENTRAL: Cochrane Central Register of Controlled Trials
CINAHL: Cumulative Index to Nursing and Allied Health Literature
GRADE: Grading of Recommendations Assessment, Development, and Evaluation
I^2^: I-squared statistic (measures between-study heterogeneity in meta-analysis)
ID: Identifier
JBI: Joanna Briggs Institute
LLRL: Low-level red-light
MEDLINE: Medical Literature Analysis and Retrieval System Online
NHLBI: National Heart, Lung, and Blood Institute
PICO: Population, Intervention, Comparator, Outcome
PICOS: Population, Intervention, Comparator, Outcomes, Study design
PRISMA: Preferred Reporting Items for Systematic Reviews and Meta-Analyses
PRISMA-P: Preferred Reporting Items for Systematic Review and Meta-Analysis Protocols
PROSPERO: International Prospective Register of Systematic Reviews
RCT: Randomized Controlled Trials
RoB 2: Cochrane Risk of Bias 2
ROBINS-I: Risk of Bias in Non-randomized Studies of Interventions
SER: Spherical Equivalent Refraction.
