# Supplementary material for: Spectral characteristics of light and their association with myopia progression in children: A systematic review protocol
Source: PLoS One. 2026 Aug 20;21(8):e0355383. doi: 10.1371/journal.pone.0355383 (PMC13492782; doi:10.1371/journal.pone.0355383)
Supplement: Supplementary file 2 [file pone.0355383.s002.pdf]

S2 Table: Timeline and status of review (CRD420251171027)

Activity/Task	Start Date	End Date	Duration	Status
Protocol Design	November 1, 2025	November 30, 2025	1 month	Completed
Search Strategy Optimization	November 1, 2025	November 30, 2025	1 month	Completed
PROSPERO Registration	November 1, 2025	November 30, 2025	1 month	Completed
Literature Search Execution	January 1, 2026	April 30, 2026	4 months	Optimizing
Record Screening (Title/Abstract & Full-Text)	August 1, 2026	October 31, 2026	3 months	Planned
Data Extraction	October 1, 2026	October 31, 2026	1 month	Planned
Quality Assessment/Risk of Bias	November 1, 2026	November 30, 2026	1 month	Planned
Final Synthesis, Meta-Analysis, and Results Reporting	November 1, 2026	December 31, 2026	2 months	Planned
